# The association between chronic obstructive pulmonary disease and autoimmune diseases: a bidirectional Mendelian randomization study

**DOI:** 10.3389/fmed.2024.1331111

**Published:** 2024-03-05

**Authors:** Xiaohui Yu, Xue Cheng, Lin Lv, Na Wang, Mengcong Li, Wenwen Ji, Tingting Liu, Guangdong Wang, Tinghua Hu, Zhihong Shi

**Affiliations:** Department of Respiratory and Critical Medicine, The First Affiliated Hospital of Xi’an Jiaotong University, Xi’an, Shaanxi, China

**Keywords:** COPD, autoimmune diseases, Mendelian randomization, causality, genome-wide association study

## Abstract

**Objective:**

Observational studies have reported that chronic obstructive pulmonary disease (COPD) is often accompanied by autoimmune diseases, but the causal relationships between them remain uncertain. In this Mendelian study, we aimed to investigate the potential causal relationship between COPD and four common autoimmune diseases.

**Methods:**

We conducted an analysis of summary data on COPD and autoimmune disease using publicly available genome-wide association studies (GWAS) summary data. We initially employed the inverse- variance weighted method as the primary approach to establish the causal impact of COPD on autoimmune diseases in the sample and conducted additional sensitivity analyses to examine the robustness of the results. Subsequently, we performed reverse Mendelian randomization (MR) analyses for the four autoimmune diseases. Finally, the potential for bidirectional causal relationships was assessed.

**Results:**

Our MR analysis revealed no significant causal relationship between COPD and any of the studied autoimmune diseases. However, reverse MR results indicated a significant association between rheumatoid arthritis (RA), osteoarthritis (OA) and the risk of developing COPD, with respective odds ratios (OR) of 377.313 (95% CI, 6.625–21487.932, *P* = 0.004) for RA and 11.097 (95% CI, 1.583–77.796, *P* = 0.015) for OA. Sensitivity analyses confirmed the robustness of the results.

**Conclusion:**

Our findings support a potential causal relationship between autoimmune diseases and COPD, highlighting the importance of considering comorbidities in clinical management of COPD.

## 1 Introduction

Chronic Obstructive Pulmonary Disease (COPD) is a heterogeneous lung condition characterized by chronic respiratory symptoms (dyspnea, cough, sputum production) due to abnormalities of the airways (bronchitis, bronchiolitis) and/or alveoli (emphysema) that cause persistent, often progressive, airflow obstruction (1). It has evolved into a global health concern, based on the definition of GOLD, the global prevalence rate of COPD among people aged 30–79 in 2019 is 10.3%, about 392 million individuals (2). A 2018 study on adult lung health in China reported an overall COPD prevalence of about 8.6% in the adult population, with a significantly higher rate of 13.7% in adults aged 40 and older (3). The presence of comorbidities, such as autoimmune diseases, significantly exacerbates the course of COPD, yet the management of COPD comorbidities has not received adequate attention in clinical practice. Previous clinical investigations have indicated a reciprocal relationship between autoimmune diseases and the development of COPD, emphasizing the need for a comprehensive understanding of the causal relationship between COPD and autoimmune diseases to improve clinical diagnostics and treatment.

Autoimmune diseases refer to chronic diseases caused by the immune system’s response to self-tissue cells, resulting in cell damage or tissue injury. The pathogenesis of autoimmune diseases is complex. The overall incidence rate accounts for approximately 3–5% of the world’s population, with approximately 40 million people in China suffering from this disease. Female patients outnumber male patients, and autoimmune diseases have a high rate of disability and mortality (4). Although both COPD and autoimmune diseases are common, their co-occurrence has not received sufficient attention. Often, patients with autoimmune diseases who take immunosuppressive drugs to control their condition are more susceptible to infection, which can lead to acute exacerbations of COPD, ultimately imposing a heavy economic and social burden (5–7).

Mendelian randomization (MR) studies are increasingly being used to infer causal relationships between risk factors and disease outcomes. Observational studies are subject to confounding factors and reverse causality, while randomized controlled trials (RCTs) require extensive human resources and follow-up time. MR studies, however, can effectively exclude confounding factors and determine the causal relationship of specific outcomes, as genetic variations are randomly assigned at conception before the onset of disease. Currently, no study has used MR methods to investigate the causal relationship between autoimmune diseases and COPD. This study can provide theoretical support for the development and progression of COPD combined with autoimmune diseases, as well as for the pathogenesis, early detection, and prevention of autoimmune diseases with COPD.

## 2 Materials and methods

### 2.1 Data sources and study design

**FIGURE 1:**
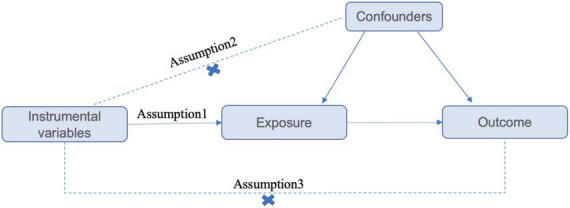
Assumptions and study design flowchart of the MR study. The MR method is based on 3 hypotheses: (1) instrumental variables directly affect the exposure; (2) instrumental variables are independent of any confounding factor; and (3) instrumental variables affect the results only via exposure but not through other pathways.

The GWAS data used in this magnetic resonance analysis are summarized statistical data obtained from published studies. The Assumptions and study design flowchart of the study is as Figure 1.

The FinnGen database^1^ was used for COPD summary data, including 18,266 cases and 311,286 controls, primarily of European descent.

The IEU database^2^ was used for autoimmune disease summary data, including 442 cases and 218,254 controls for systemic lupus erythematosus (SLE), 3,730 cases and 333,429 controls for rheumatoid arthritis (RA), 12,882 cases and 21,770 controls for inflammatory bowel disease (IBD), and 28,257 cases and 308,902 controls for osteoarthritis (OA). All samples were from individuals of European descent. Refer to Table 1.

**TABLE 1:** A summary of the data for Mendelian randomization analysis.

Diseases	ID	Cases	Controls	Year	Number of SNPs
COPD	finn-b-J10_COPD	18266	311286	2023	20169090
SLE	finn-b-SLE_NOS	442	218254	2021	16380466
RA	ukb-a-105	3730	333429	2018	10894596
IBD	ieu-a-31	12882	21770	2015	12716084
OA	ukb-a-106	28257	308902	2017	10894596

### 2.2 Instrumental variable selection

To satisfy the first MR assumption, namely that the single nucleotide polymorphism (SNP) must be closely associated with the exposure factor, SNPs significantly associated with the exposure were selected at the genome-wide level (*P* < 5 × 10^^–^8, *r*^2^ < 0.001, genetic distance = 10,000 kb). If there were too few included SNPs, the criteria could be adjusted to (*P* < 5 × 10^^–^6, *r*^2^ < 0.001, genetic distance = 10,000 kb).

To ensure that the genetic variation is unrelated to potential confounders, which is the second MR assumption, a query was conducted in the Phenoscanner database to confirm that the included SNPs were unrelated to known confounders. In other words, instrumental variables should not influence the outcome through any other pathway besides exposure.

To meet the third MR assumption, this study employed the MR-Egger intercept test to examine the presence of horizontal pleiotropy between SNPs and the outcome factor (8). When the intercept of the regression line is non-zero, it indicates the presence of horizontal pleiotropy between instrumental variables and the outcome. Conversely, if the intercept is zero, there is no horizontal pleiotropy. Additionally, SNPs with palindromic alleles in the GWAS were excluded in the final calculation. Ultimately, SNPs significantly associated with the exposure factor were obtained as instrumental variables.

The F-statistic for instrumental variables was calculated to assess the strength of the instrumental variables (IVs) chosen in relation to the exposure. *R*^2^ represents the variance explained by the genetic instruments and is computed using the formula:

*R*^2^ = β^2^(1−*EAF*) × 2*EAF*, where EAF is the allele frequency of the mutant gene. The F-statistic is calculated using the formula:

$F=R^{2}\,\frac{\left(N-K-1\right)}{K\,\left(1-R^{2}\right)}$ (8, 9). Here, K is the number of SNP-exposure associations, and N is the sample size of the GWAS SNP-exposure association. SNPs with an F-statistic >10 were defined as reliable and effective IVs, which helps prevent weak instrument bias from affecting MR results (10).

### 2.3 Statistical analysis

In this study, two-sample MR analyses were primarily conducted using the Inverse Variance Weighted (IVW) method. In the absence of heterogeneity, a fixed-effects model was employed, while a random-effects model was used in the presence of heterogeneity (11). Additionally, MR-Egger regression and the Weighted Median method (WM) were utilized to complement the IVW results. Conventional inverse variance weighting analysis methods may be influenced by weak instrument bias or pleiotropy. Therefore, this study employed sensitivity analysis to assess the validity and robustness of IVW results. As the outcomes were binary variables, effect estimates were further transformed into OR to provide a more intuitive understanding of the relationship between exposure and outcome.

### 2.4 Sensitivity analysis

Various sensitivity analysis methods were employed in this study to ensure the stability of the results. First, Cochran’s *Q*-test assessed heterogeneity among individual SNP estimates and provided evidence for selecting the appropriate analysis method. If the *p*-value was greater than 0.05, indicating no heterogeneity, the fixed-effects IVW method was considered the primary approach; otherwise, a random-effects model was used. Secondly, the MR-Egger intercept method was used to detect horizontal pleiotropy of IVs. In MR-Egger testing, the intercept estimated the overall average horizontal pleiotropic effect of all SNPs. If the *p*-value was less than 0.05, there might be bias in IVW estimates. Additionally, MR-PRESSO was used to identify the presence of outliers and exclude them from the analysis (12). Finally, we conducted a leave-one-out sensitivity test to evaluate the influence of individual SNPs on causal effects. Furthermore, funnel plots and forest plots were generated to detect the presence of pleiotropy.

Statistical analysis was performed using the “TwoSample MR” and “MR-PRESSO” packages in R 4.2.0 software. All statistical tests were two-tailed, and significance was set at *P* < 0.05.

## 3 Result

### 3.1 Causal impact of COPD on autoimmune diseases

In this study, a total of 13 SNPs strongly associated with COPD were included. After excluding SNPs potentially related to confounding factors and palindromic SNPs, 11, 10, 10, and 10 SNPs were, respectively, selected for Mendelian randomization (MR) analyses concerning Systemic Lupus Erythematosus (SLE), Rheumatoid Arthritis (RA), Inflammatory Bowel Disease (IBD), and Osteoarthritis (OA). The F-statistics for all SNPs exceeded 10, indicating that the estimates were unlikely to be influenced by weak instrumental variable bias refer to Supplementary Table 1 for details.

**FIGURE 2:**
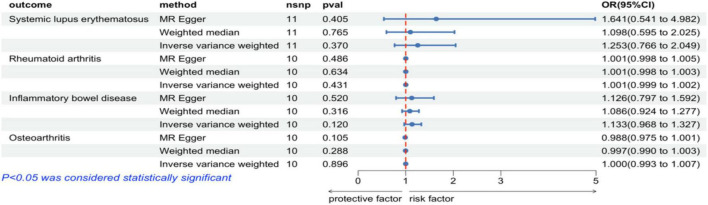
Risk estimates of COPD for four autoimmune diseases.

Results of MR Analysis are Presented in Figure 2 and Supplementary Table 2. The findings indicate that in the IVW analysis, the genetically predicted COPD is not associated with the risk of any of the four autoimmune diseases. The results are consistent in the MR-Egger and WM analyses, and the MR-PRESSO analysis did not detect any outliers, which suggests that the results are robust and reliable (Supplementary Table 2). There was no evidence of heterogeneity among individual SNP estimates in the Cochran’s *Q*-test (*Q* = 11.388; *P* = 0.273), and no pleiotropy was observed in the MR-Egger regression analysis (Intercept = −0.034; *P* = 0.604; Supplementary Table 2). Furthermore, leave-one-out analysis demonstrates that the causal impact of COPD on the four autoimmune diseases is not driven by any single SNP (Supplementary Figure 1).

### 3.2 Causal impact of autoimmune diseases on COPD

In the reverse Mendelian randomization (MR) analysis investigating the causal impact of autoimmune diseases on COPD, we opted for 13 SNPs for SLE, 4 SNPs for RA, 49 SNPs for IBD, and 27 SNPs for OA as IVs, following the exclusion of palindromic and uninformative SNPs. All the selected SNPs exhibited F-statistics exceeding 10 to mitigate the potential bias stemming from weak instruments (Supplementary Table 3).

**FIGURE 3:**
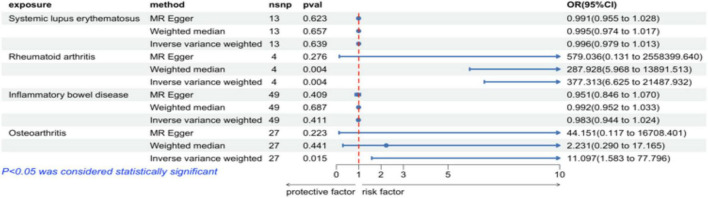
Risk estimates of four autoimmune diseases for COPD.

**FIGURE 4:**
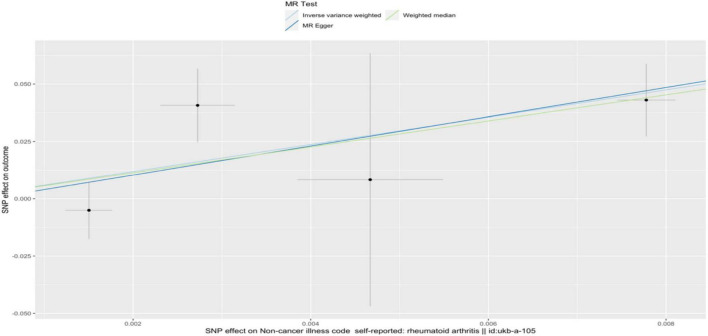
A scatterplot used to analyze the genetic causal relationship between RA and COPD. In the scatterplot, the *x*-axis represents the effect of individual SNPs on RA, and the *y*-axis represents the effect of individual SNPs on COPD. The colored diagonal line represents the fitted line for the causal effect estimation of RA on COPD. From the graph, it can be observed that as the risk of RA increases, the risk of COPD incidence also rises.

**FIGURE 5:**
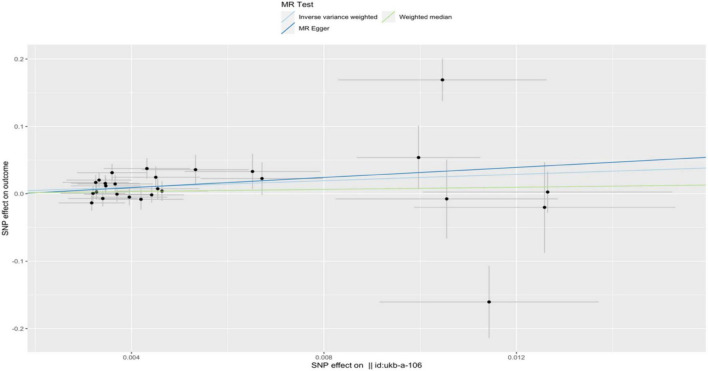
A scatterplot used to analyze the genetic causal relationship between OA and COPD. In the scatterplot, the *x*-axis represents the effect of individual SNPs on OA, and the *y*-axis represents the effect of individual SNPs on COPD. The colored diagonal line represents the fitted line for the causal effect estimation of OA on COPD. From the graph, it can be observed that as the risk of OA increases, the risk of COPD incidence also rises.

In the IVW analysis, we observed a positive association between the genetic susceptibility of RA and OA with COPD. The odds ratios (OR) for RA and OA were 377.313 (95% CI, 6.625–21487.932; *P* = 0.004) (Figures 2, 3 and Supplementary Table 4) And 11.097 (95% CI, 1.583–77.796; *P* = 0.015), respectively. Although the statistical significance was not pronounced in the MR-Egger method for the causal impact of OA on COPD and in the MR-Egger and WM methods for the causal impact of RA on COPD, these methods exhibited similar trends to the primary IVW method. Therefore, we consider these results reliable (Figures 4, 5 and Supplementary Table 4).

### 3.3 Sensitivity analysis

In sensitivity analysis, Cochran’s *Q*-test revealed significant heterogeneity in IBD and OA (PIBD = 1.62E-11; POA = 0.0003) (Supplementary Table 3). Consequently, the IVW random-effects model was employed for the analysis. MR-PRESSO analysis did not detect any outliers in SLE, RA, IBD, or OA (Supplementary Table 3). Furthermore, leave-one-out analysis indicated that the causal relationship between SLE, RA, IBD, or OA and COPD is not driven by any single SNP. MR funnel plots, leave-one-out analysis, and scatterplots can be found in Supplementary Figure 2.

## 4 Discussion

In this study, we conducted the first two-sample bidirectional MR analysis to investigate the potential causal relationship between COPD and autoimmune diseases. The results indicate no evidence of an association between COPD and SLE, RA, IBD, or OA. However, reverse MR analysis revealed a significant positive correlation between the genetic predisposition to RA and OA and an increased risk of COPD. Furthermore, the genetic susceptibility of SLE and IBD exhibited a negative association with COPD risk, although it was not statistically significant.

Obstructive pulmonary disease is characterized by persistent respiratory symptoms and airflow limitation, resulting from chronic airway inflammation, tissue destruction, and airflow obstruction. Increasing evidence suggests that autoimmune pathogenesis may play a vital role in the occurrence and development of COPD (13).

Observational studies have found a high correlation between RA and interstitial lung disease, as well as COPD. The incidence of COPD in RA patients has been reported to be significantly high in various studies (14), with a pooled analysis showing a COPD prevalence of 6.2% in RA (15). While smoking is considered a risk factor for both RA and COPD, studies have shown a high prevalence of COPD in RA patients even after adjusting for smoking (16, 17). Observational studies have also demonstrated a significant association between RA and the subsequent development of COPD (18), consistent with our findings of a causal effect of RA on COPD. Currently, there is no concrete evidence to explain the specific interplay between the development of both diseases when they coexist. Some studies suggest that the use of immunosuppressants is not significantly associated with the prognosis of COPD (19). However, other research suggests a correlation with the production of autoantigens and decreased immune tolerance in COPD patients, with a significant increase in serum autoantibody titers related to disease severity (20). There are also studies indicating overlapping pathogenic mechanisms between respiratory inflammation and autoimmune diseases, such as Granulomatosis with Polyangiitis patients showing false-positive COVID-19 antibody tests, possibly due to an excess of autoantibodies against cell antigens in patients with autoimmune diseases (21). Serum from COPD patients has been shown to produce autoantibodies that react with antigens known to be associated with autoimmune diseases, including RA (22). Further research is needed to investigate the potential mechanisms by which RA contributes to COPD.

A pooled analysis has shown a high prevalence of osteoarthritis (OA) in COPD patients, with an estimated overall prevalence of 35.5% for OA in COPD (23). Common risk factors between COPD and OA are believed to increase the likelihood of both diseases occurring simultaneously. The specific mechanisms by which OA functions in COPD may be related to systemic inflammatory mediators, reduced skeletal muscle function, and more (24, 25). Studies suggest that neutrophil elastase, which is associated with tissue destruction in inflammatory joint diseases, also plays a significant role in the pathogenesis of COPD (26).

Research indicates an increased risk of COPD in SLE patients (7). A large population study from Taiwan showed that the overall prevalence of chronic obstructive pulmonary disease was 1.73 times higher in SLE patients than in the control group (7). Population-based studies have found an increased incidence of IBD in COPD patients (27). However, these findings are inconsistent with our study results. The inconsistency may be due to the susceptibility of observational studies to the influence of confounding factors.

In conclusion, this bidirectional MR study investigated the causal relationship between COPD and autoimmune diseases, as well as the causal impact of autoimmune diseases on COPD. Further research is needed to explore and elucidate the biological mechanisms between COPD and autoimmune diseases to better understand and manage these diseases in clinical practice. Considering the potential positive association between autoimmune diseases and the risk of COPD, it is recommended to prioritize early diagnosis and prevention of COPD in populations with autoimmune diseases to provide better prognosis and quality of life for patients.

Our study has several strengths and limitations. Firstly, this is the first study to investigate the bidirectional causal relationship between COPD and autoimmune diseases using a two-sample MR, which is less susceptible to confounding factors and reverse causation compared to observational studies. We also conducted multiple sensitivity tests and IV strength assessments to ensure the robustness and validity of the results. However, the study has some limitations. First, our research is limited to the genetic level and cannot explore more specific phenotypes, such as the severity of COPD or acute exacerbations. Further research, such as randomized controlled trials (RCTs), is needed to help identify patients with specific immune responses to airway epithelial cell damage, combined with biomarkers beneficial for identifying and assessing patient conditions, such as red cell distribution width (RDW) as a prognostic marker for COPD patients (28), to advance toward precision medicine approaches. Secondly, the impact of clinical interventions cannot be determined through Mendelian randomization analysis. Lastly, this study primarily focused on individuals of European ancestry, which limits the generalizability of our findings to other populations. While we explored the genetic aspects of the relationship between COPD and autoimmune diseases, the underlying mechanisms remain unclear and require further investigation.

## 5 Conclusion

In conclusion, this MR study indicates a potential causal relationship between RA and OA and the risk of COPD. Based on our current research findings, we believe that screening for COPD in patients with RA and OA may be meaningful.

## Data availability statement

The original contributions presented in the study are included in the article/Supplementary material, further inquiries can be directed to the corresponding author.

## Ethics statement

All data sets provided by GWAS have been approved by the ethics committee related to the original research.

## Author contributions

XY: Conceptualization, Methodology, Resources, Visualization, Writing – original draft, Writing – review and editing. XC: Data curation, Formal Analysis, Writing – review and editing. LV: Data curation, Formal Analysis, Writing – review and editing. NW: Software, Validation, Writing – review and editing. ML: Software, Validation, Writing – review and editing. WJ: Software, Validation, Writing – review and editing. TL: Software, Validation, Writing – review and editing. GW: Software, Validation, Writing – review and editing. TH: Supervision, Writing – review and editing. ZS: Funding acquisition, Supervision, Writing – review and editing.

## Footnotes

1
https://r9.finngen.fi/

2
https://gwas.mrcieu.ac.uk/
