# Supplementary material for: The association between chronic obstructive pulmonary disease and autoimmune diseases: a bidirectional Mendelian randomization study
Source: Front Med (Lausanne). 2024 Mar 5;11:1331111. doi: 10.3389/fmed.2024.1331111 (PMC10949139; doi:10.3389/fmed.2024.1331111)
Supplement: Supplementary file 2 [file Data_Sheet_1.docx]

**Supplementary Figure 1**

**Systemic lupus erythematosus**


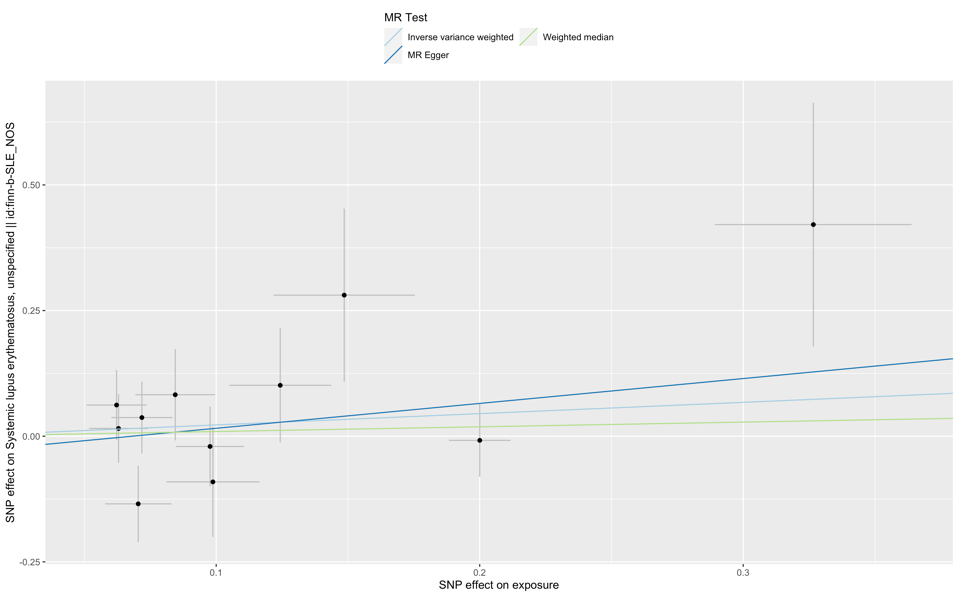


A. Scatter plot for casual effects of COPD on SLE. SNP: single nucleotide polymorphism. The slope of each line represents an estimate of the effect of a different method using MR.


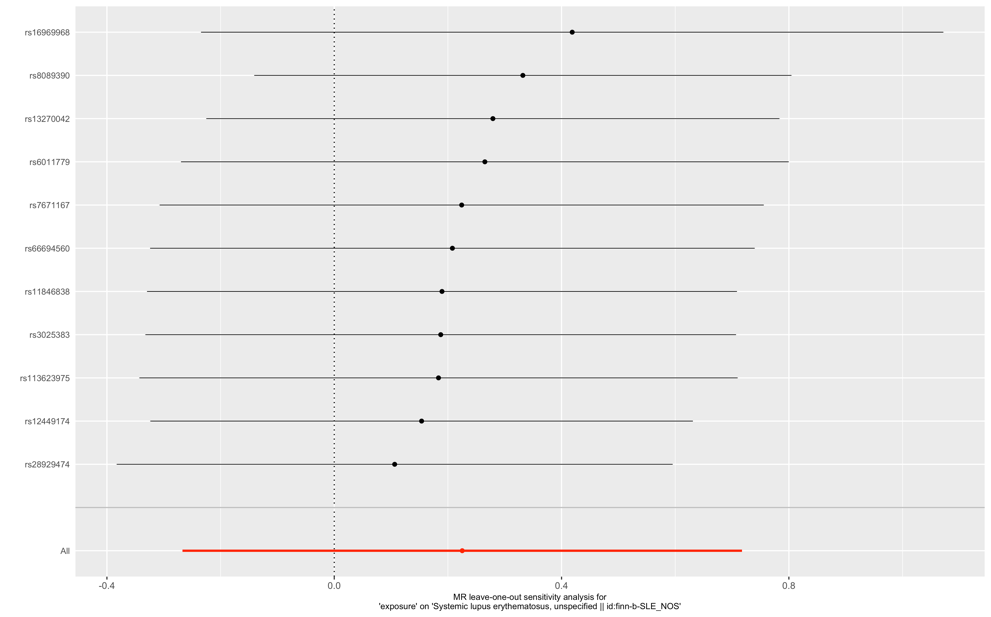


B. The leave-one-out sensitivity analysis assessed the causal association between COPD and SLE.

**
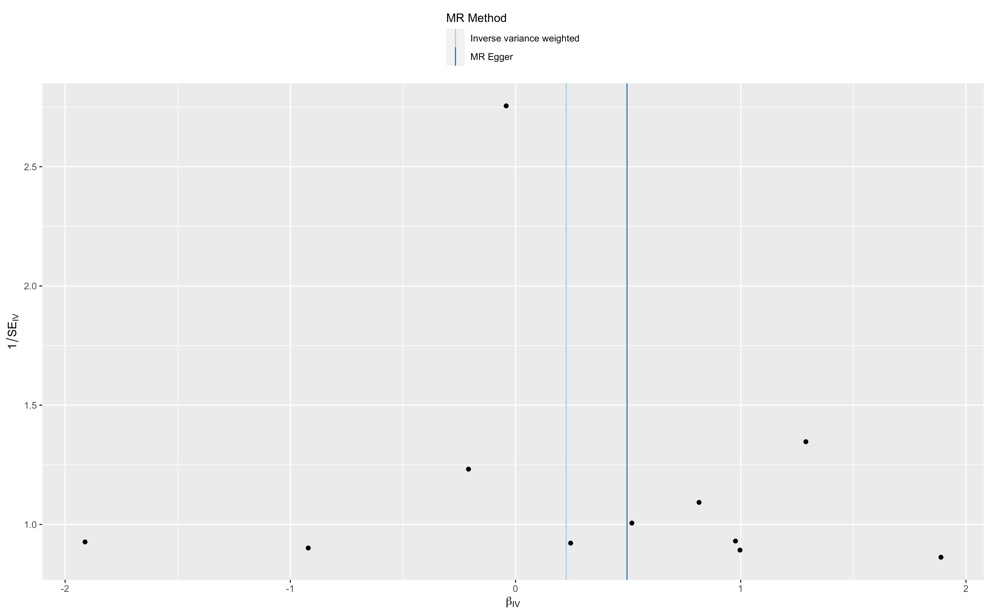
**

C. Funnel plot of causality between COPD and SLE

**Rheumatoid arthritis**


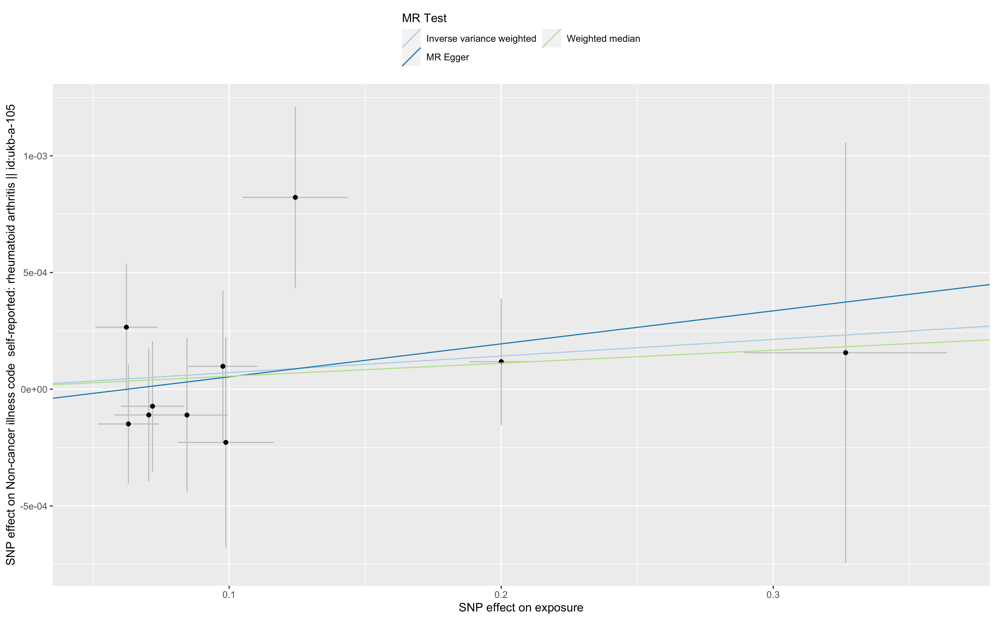


A. Scatter plot for casual effects of COPD on RA. SNP: single nucleotide polymorphism. The slope of each line represents an estimate of the effect of a different method using MR.


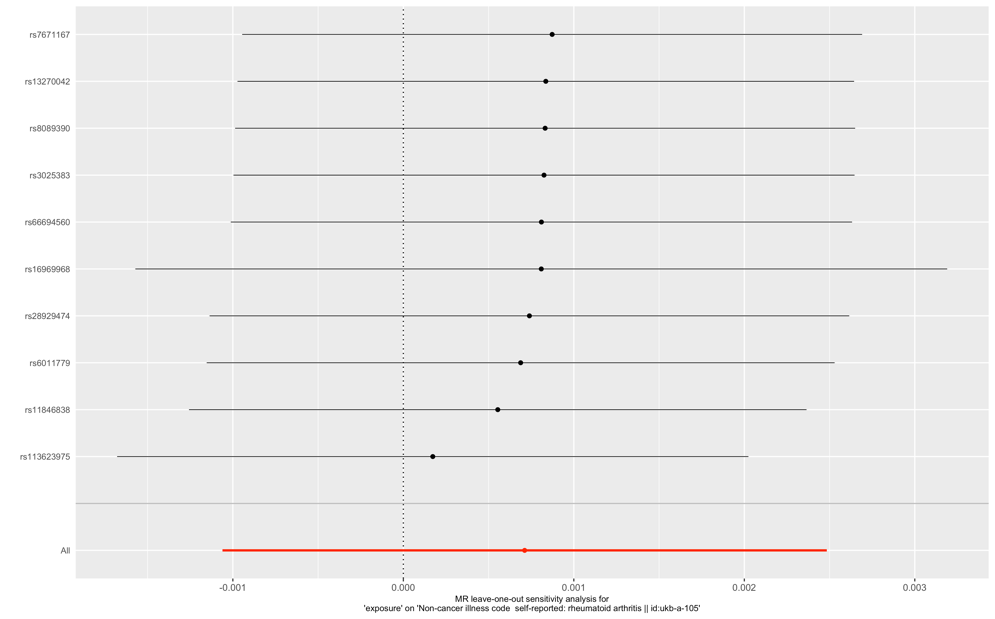


B. The leave-one-out sensitivity analysis assessed the causal association between COPD and RA.


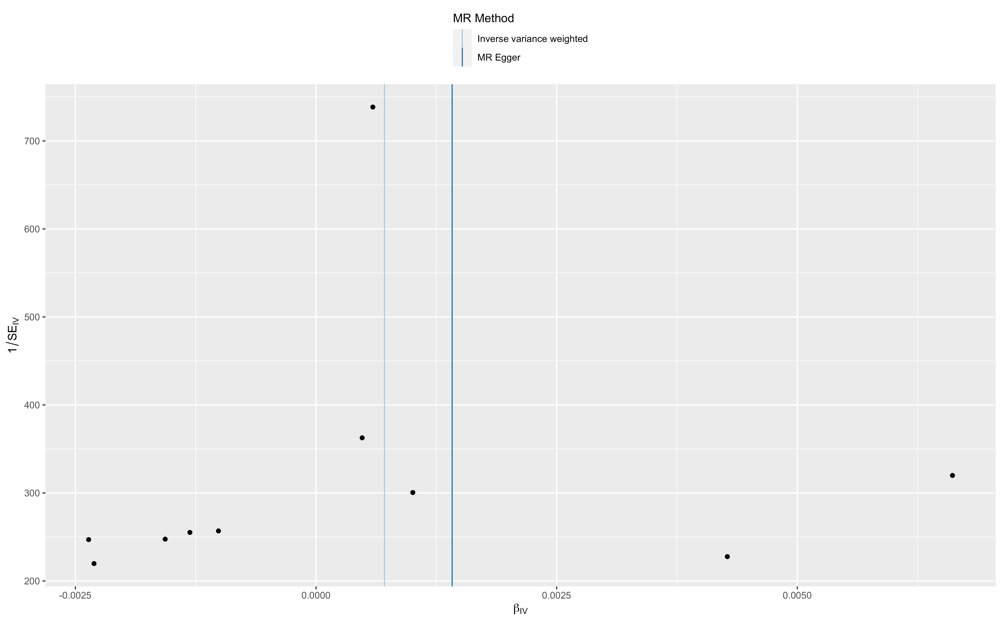


C. Funnel plot of causality between COPD and RA.

**Inflammatory bowel disease**


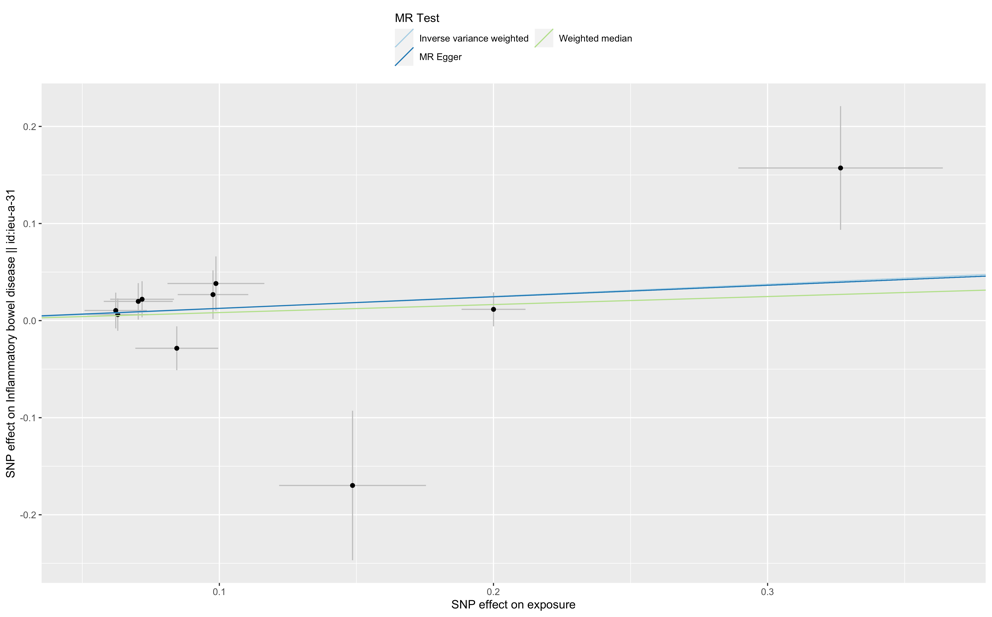


A. Scatter plot for casual effects of COPD on IBD. SNP: single nucleotide polymorphism. The slope of each line represents an estimate of the effect of a different method using MR.


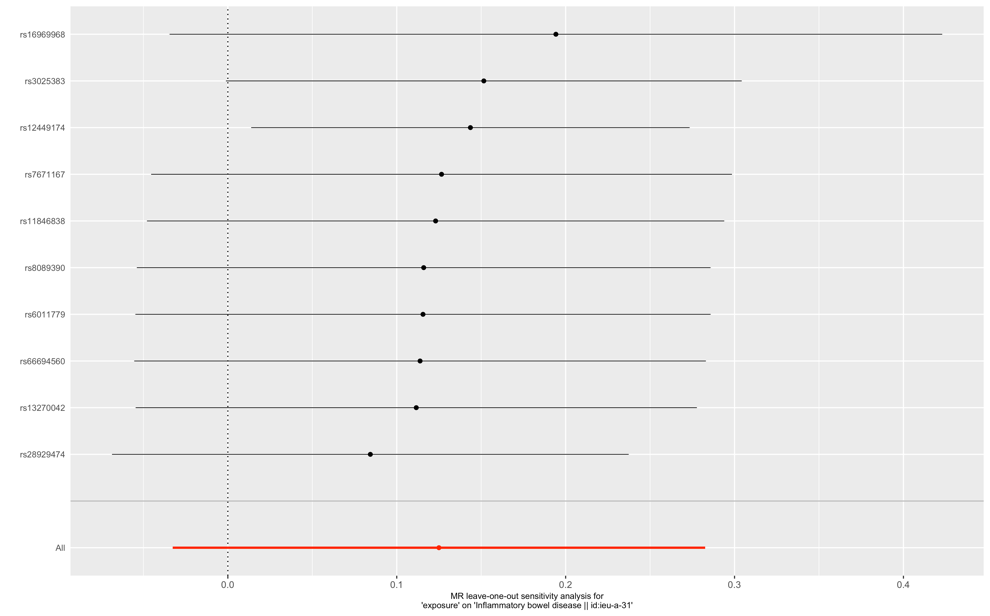


B. The leave-one-out sensitivity analysis assessed the causal association between COPD and IBD.


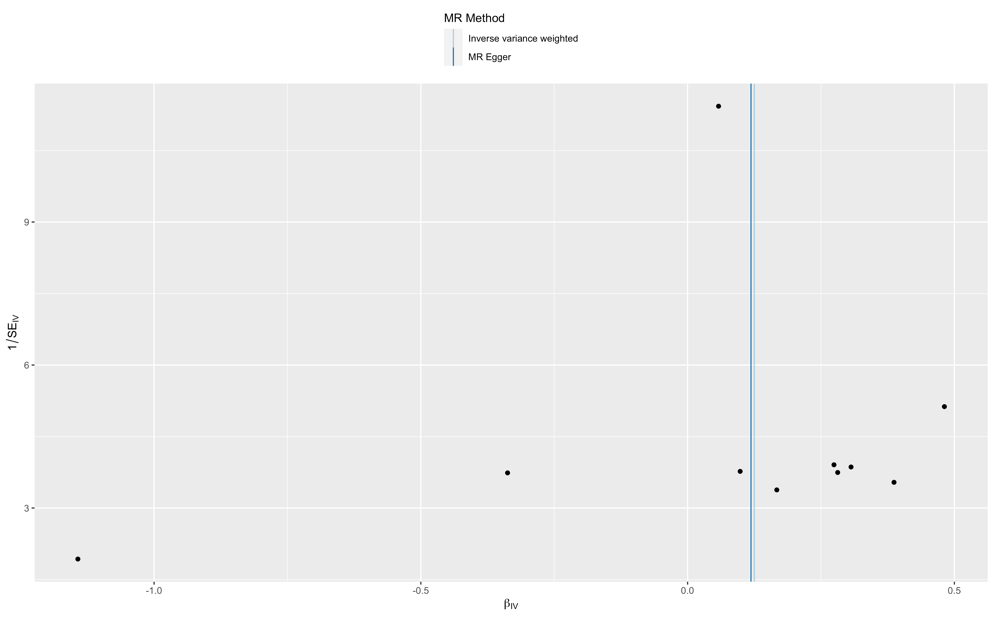


C. Funnel plot of causality between COPD and IBD

**Osteoarthritis**


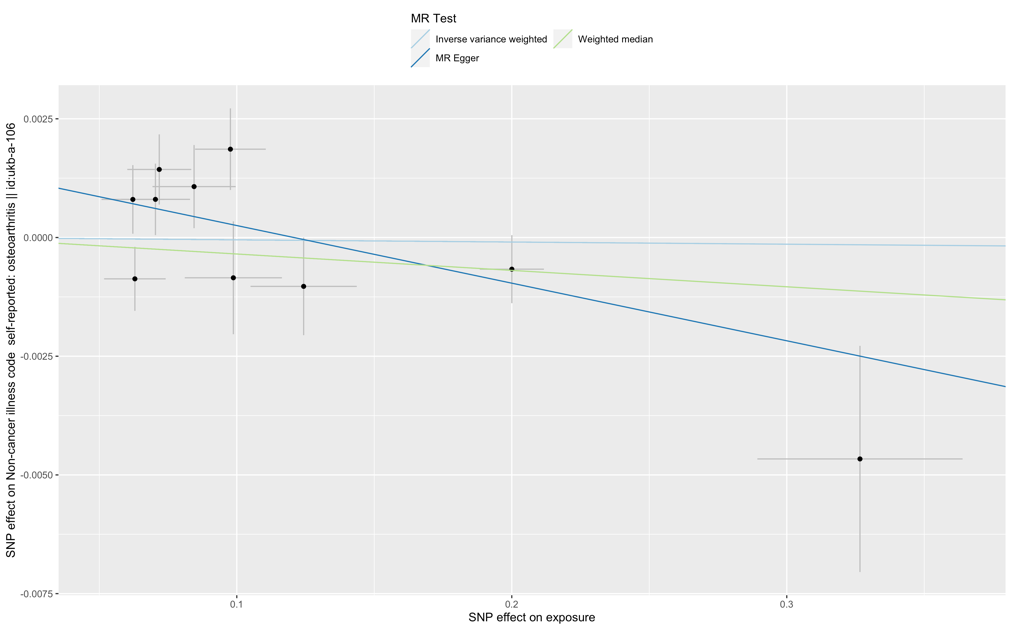


A. Scatter plot for casual effects of COPD on OA. SNP: single nucleotide polymorphism. The slope of each line represents an estimate of the effect of a different method using MR.


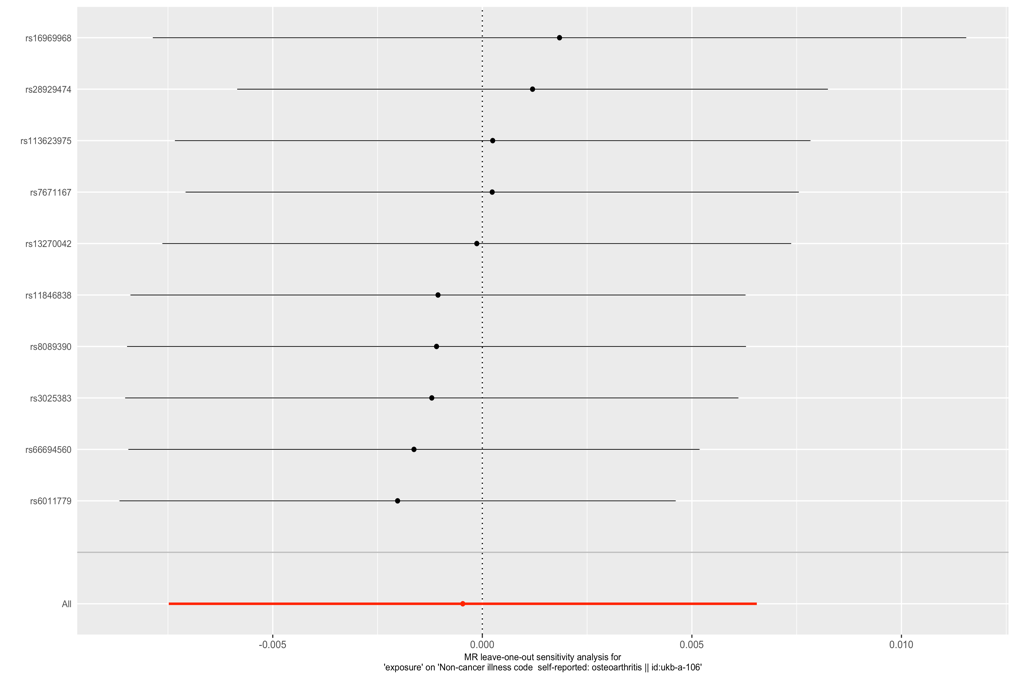


B. The leave-one-out sensitivity analysis assessed the causal association between COPD and OA.


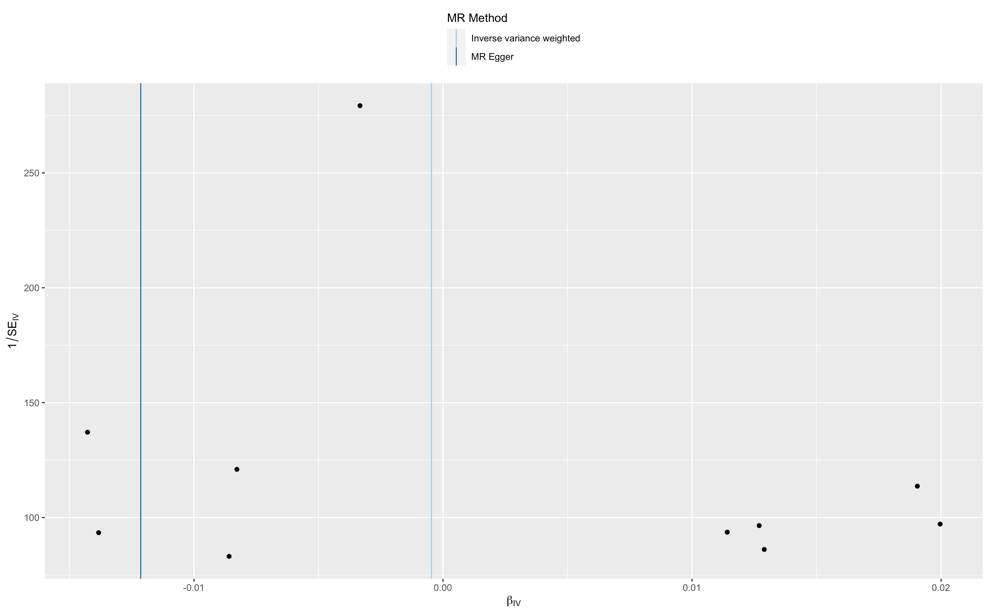


C. Funnel plot of causality between COPD and OA.
