# Supplementary material for: The association between chronic obstructive pulmonary disease and autoimmune diseases: a bidirectional Mendelian randomization study
Source: Front Med (Lausanne). 2024 Mar 5;11:1331111. doi: 10.3389/fmed.2024.1331111 (PMC10949139; doi:10.3389/fmed.2024.1331111)
Supplement: Supplementary file 3 [file Data_Sheet_2.docx]

**Supplementary Figure 2**

**Systemic lupus erythematosus**


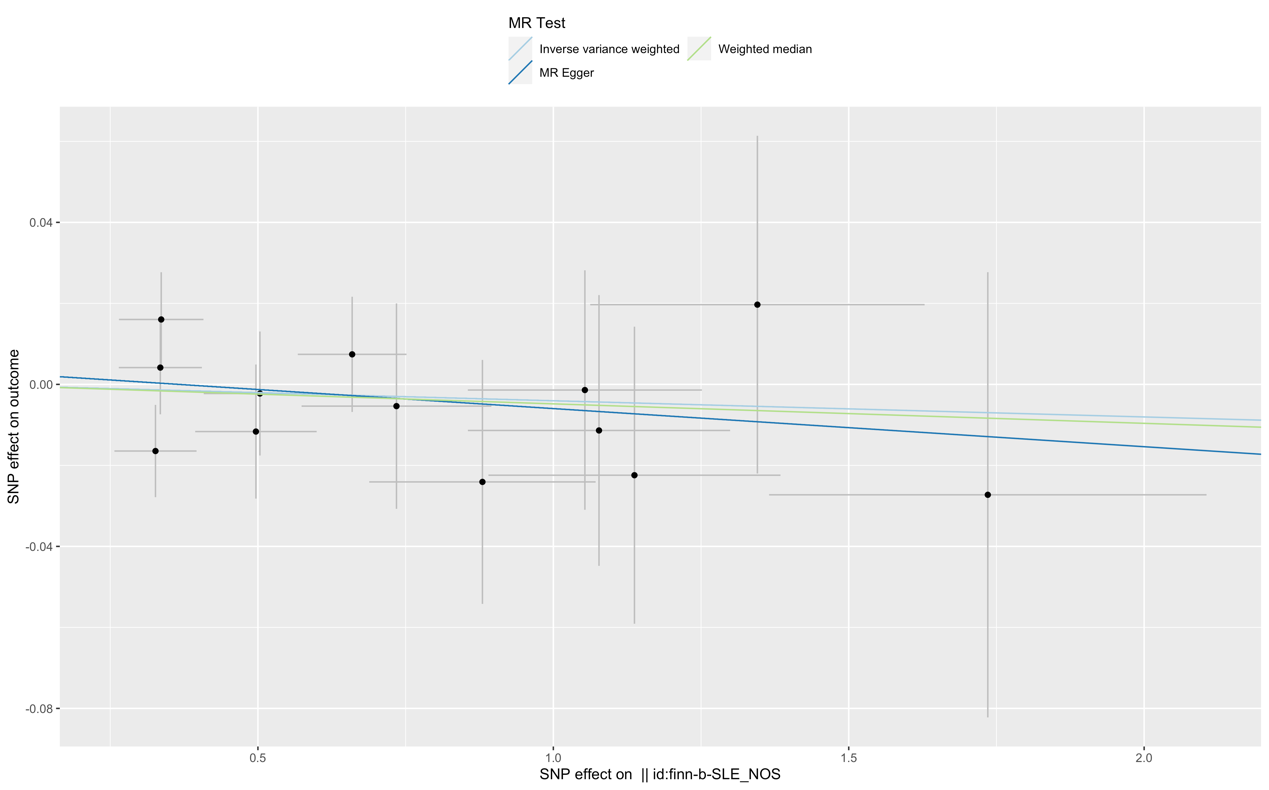


A. Scatter plot for casual effects of SLE on COPD. SNP: single nucleotide polymorphism. The slope of each line represents an estimate of the effect of a different method using MR.


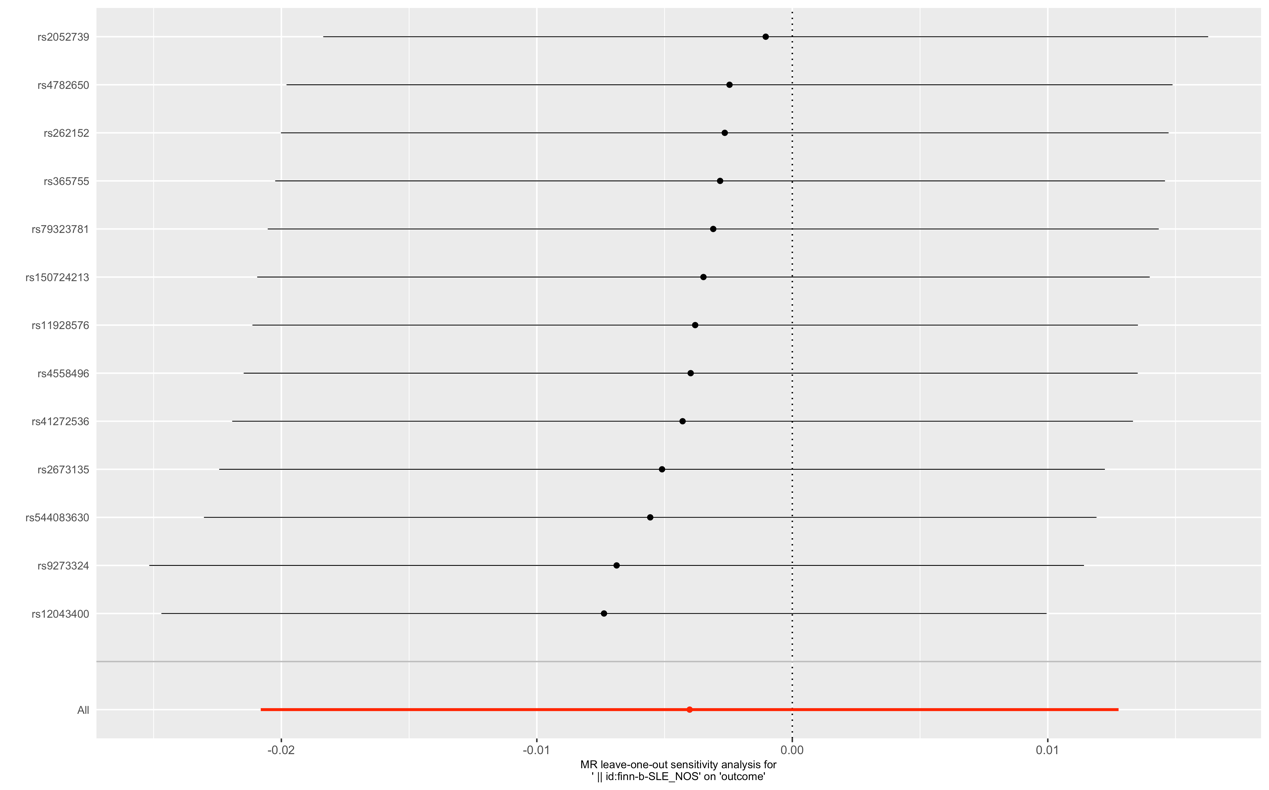


B. The leave-one-out sensitivity analysis assessed the causal association between SLE and COPD.


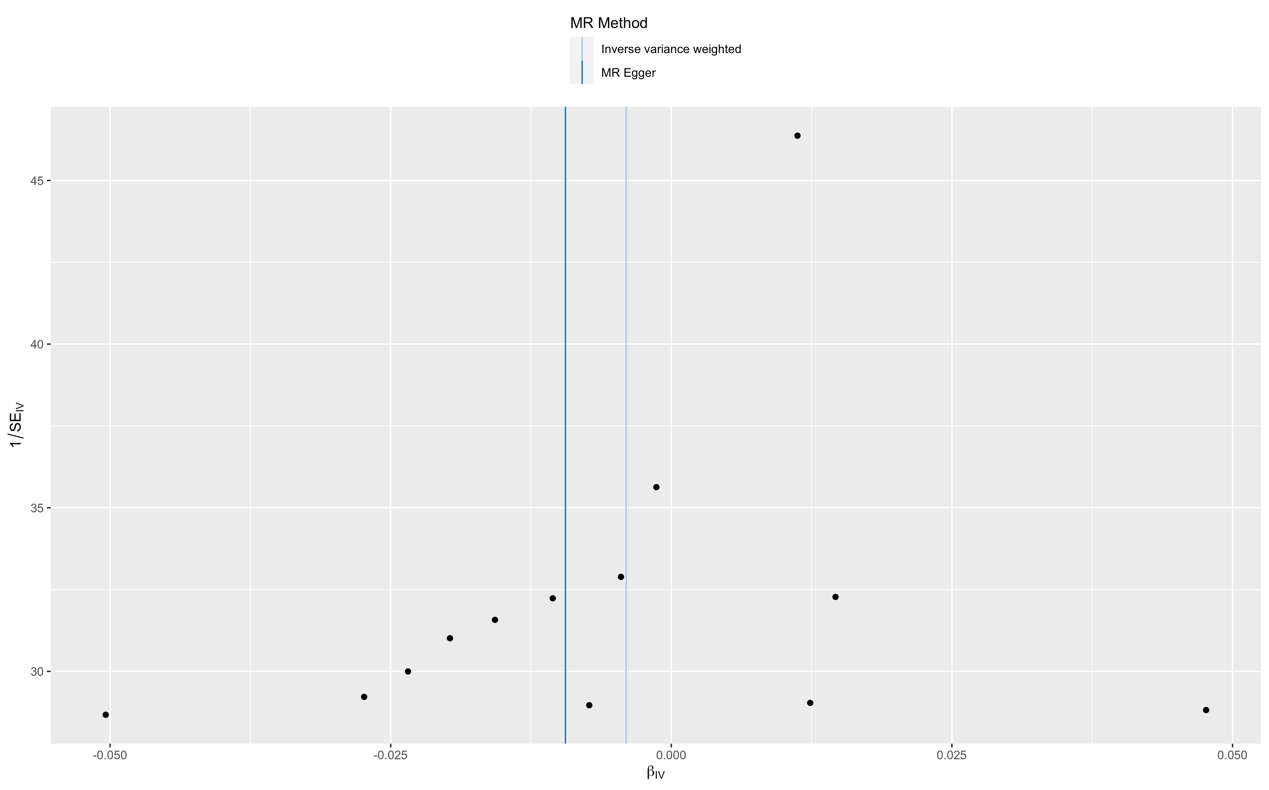


C. Funnel plot of causality between SLE and COPD.

**Rheumatoid arthritis**


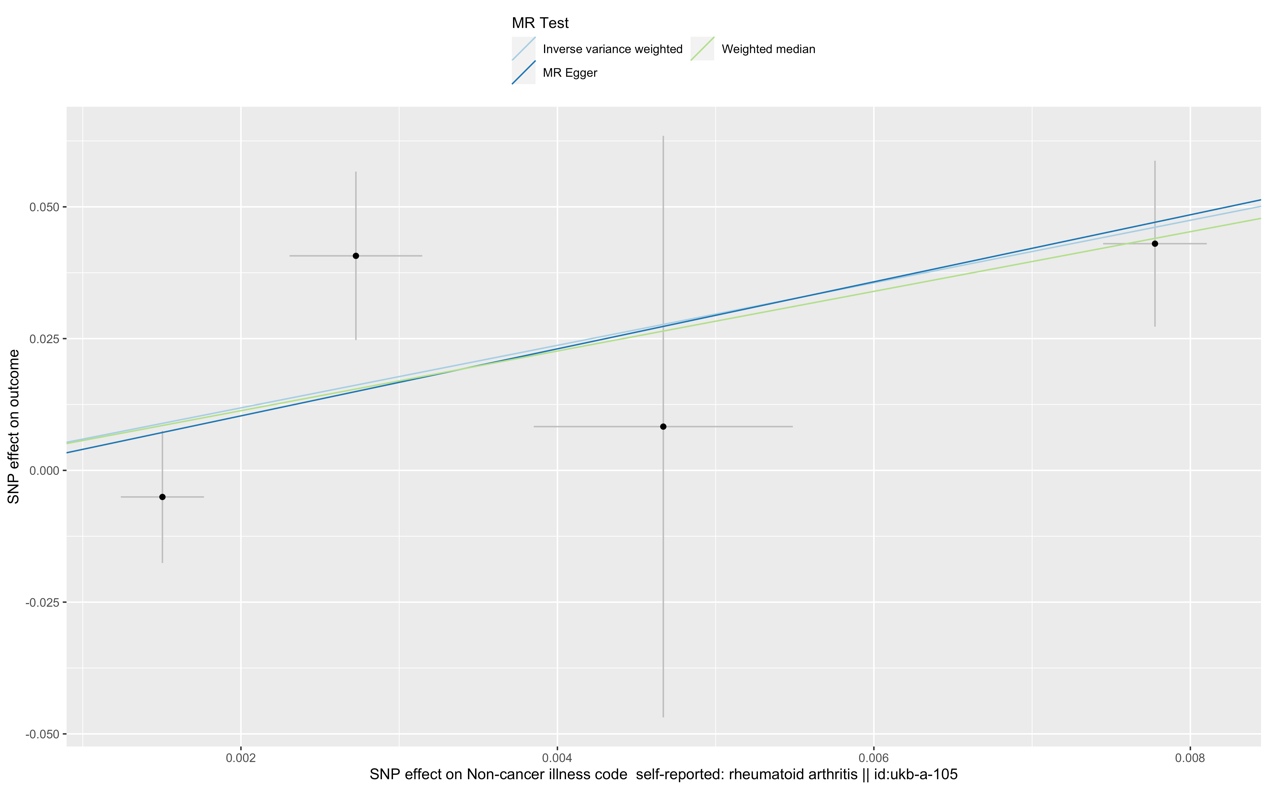


A. Scatter plot for casual effects of RA on COPD. SNP: single nucleotide polymorphism. The slope of each line represents an estimate of the effect of a different method using MR.


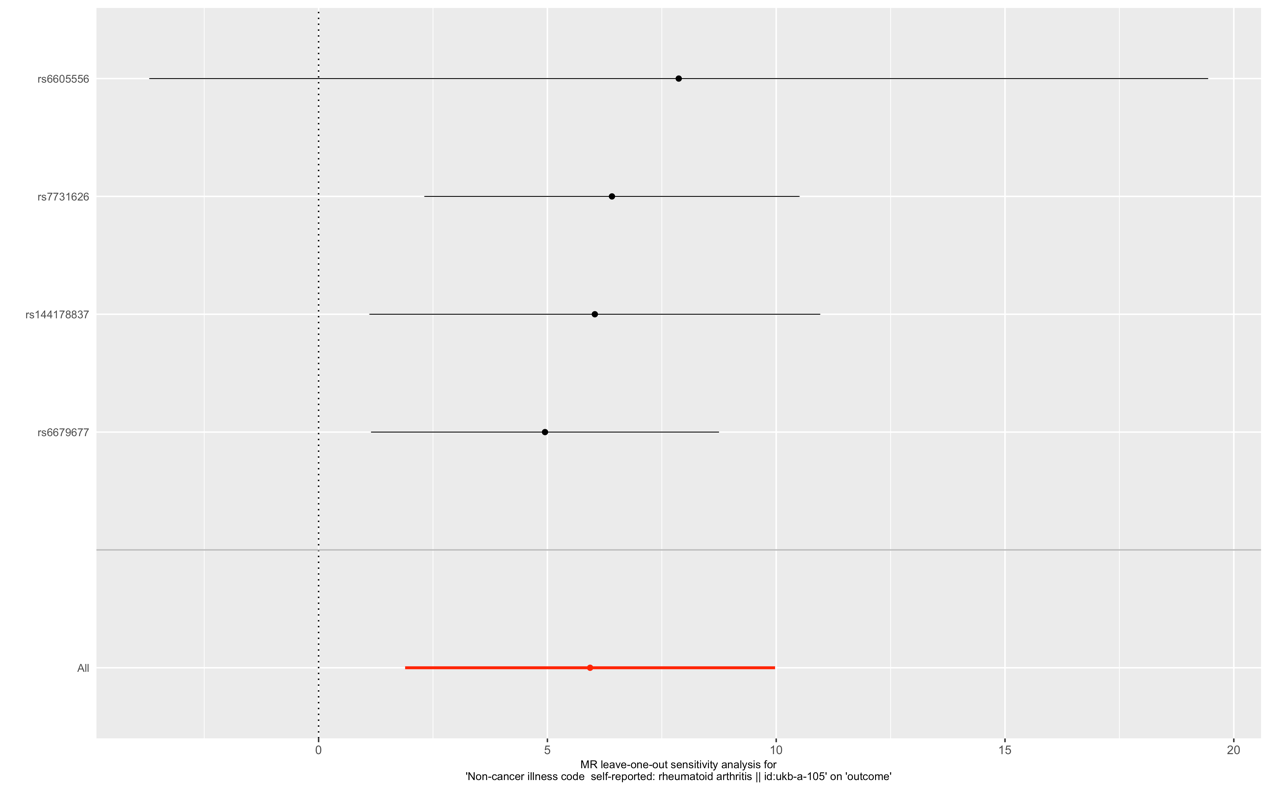


B. The leave-one-out sensitivity analysis assessed the causal association between RA and COPD.


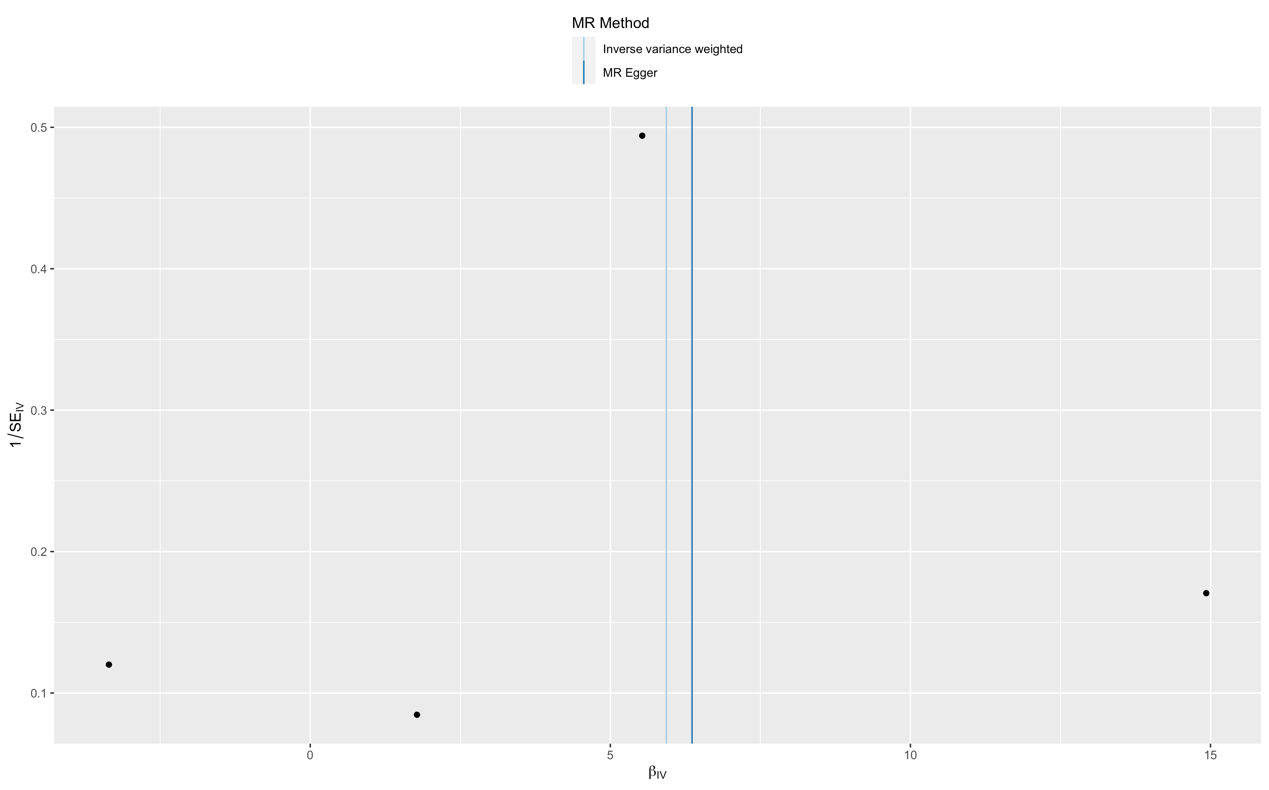


C. Funnel plot of causality between RA and COPD.
