# Supplementary material for: The association between chronic obstructive pulmonary disease and autoimmune diseases: a bidirectional Mendelian randomization study
Source: Front Med (Lausanne). 2024 Mar 5;11:1331111. doi: 10.3389/fmed.2024.1331111 (PMC10949139; doi:10.3389/fmed.2024.1331111)
Supplement: Supplementary file 4 [file Data_Sheet_3.docx]

**Supplementary Figure 3**

**Inflammatory bowel disease**


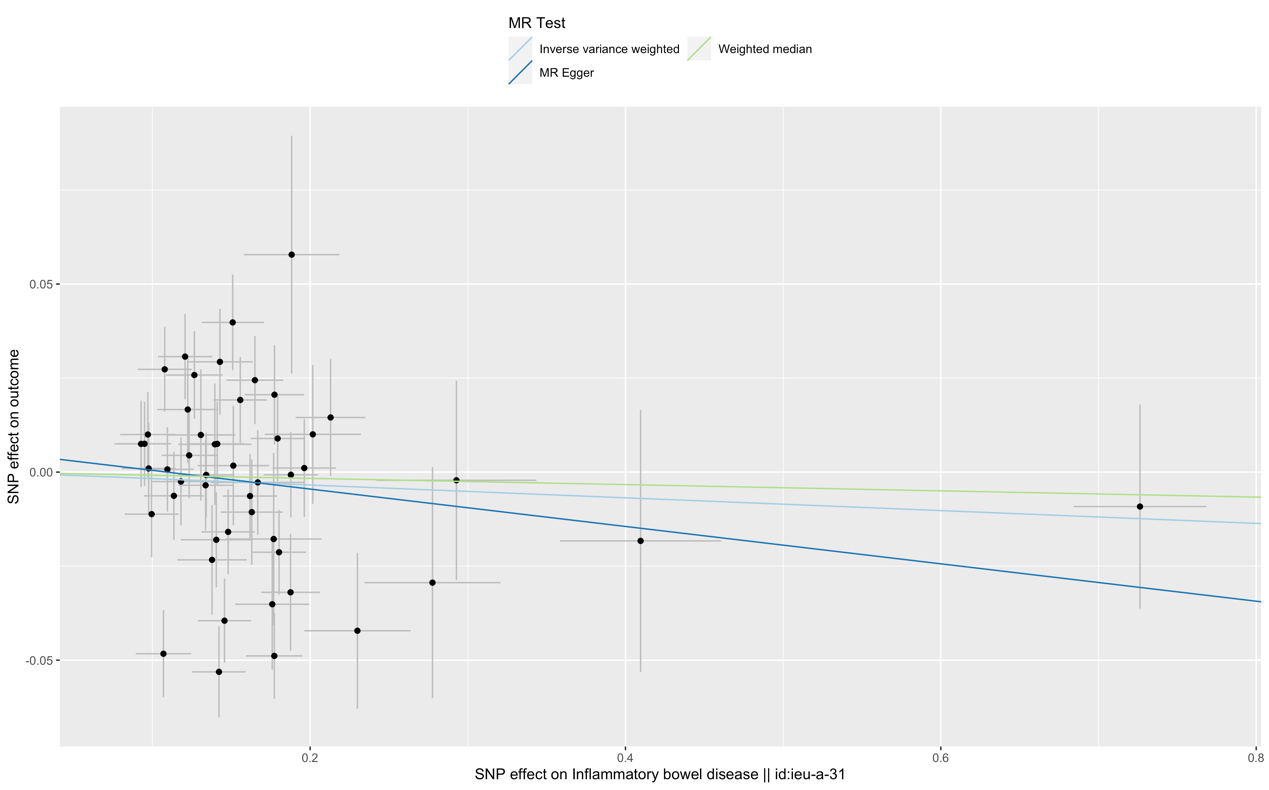


A. Scatter plot for casual effects of IBD on COPD. SNP: single nucleotide polymorphism. The slope of each line represents an estimate of the effect of a different method using MR.


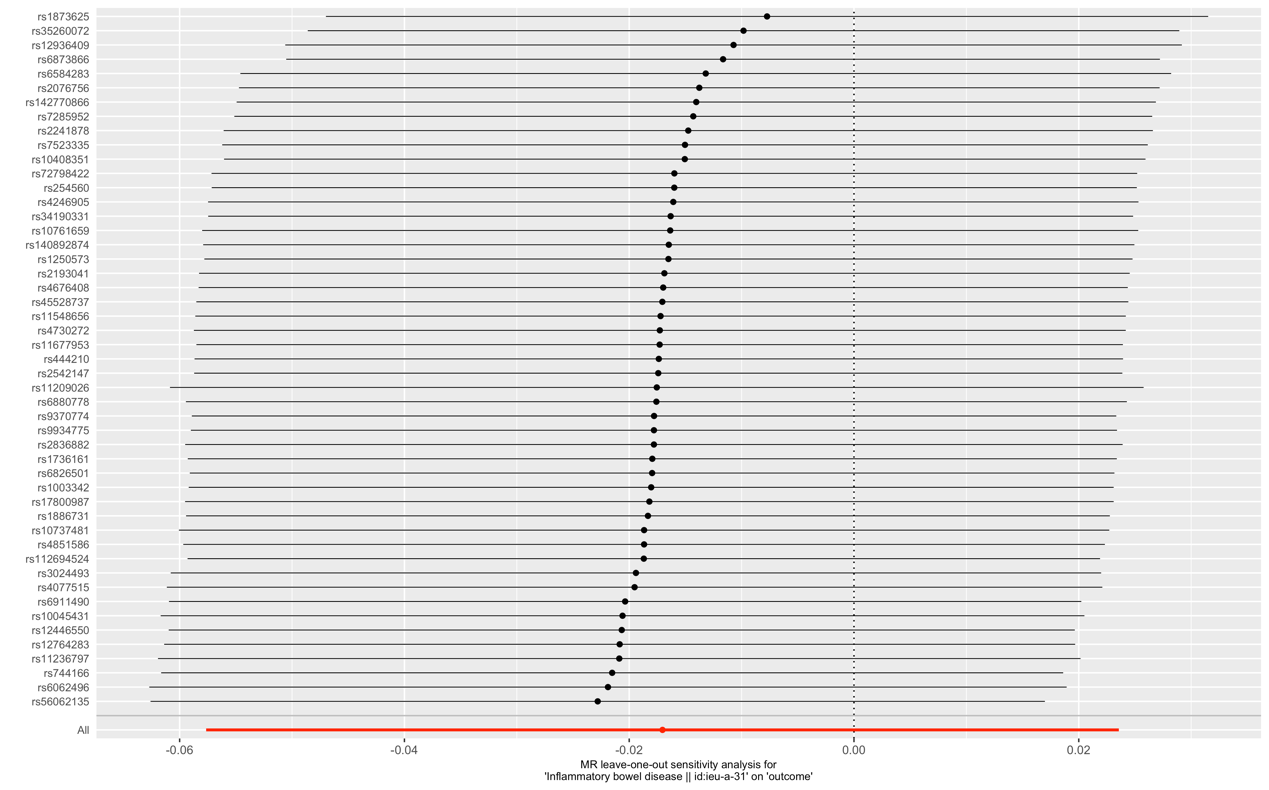


B. The leave-one-out sensitivity analysis assessed the causal association between IBD and COPD.


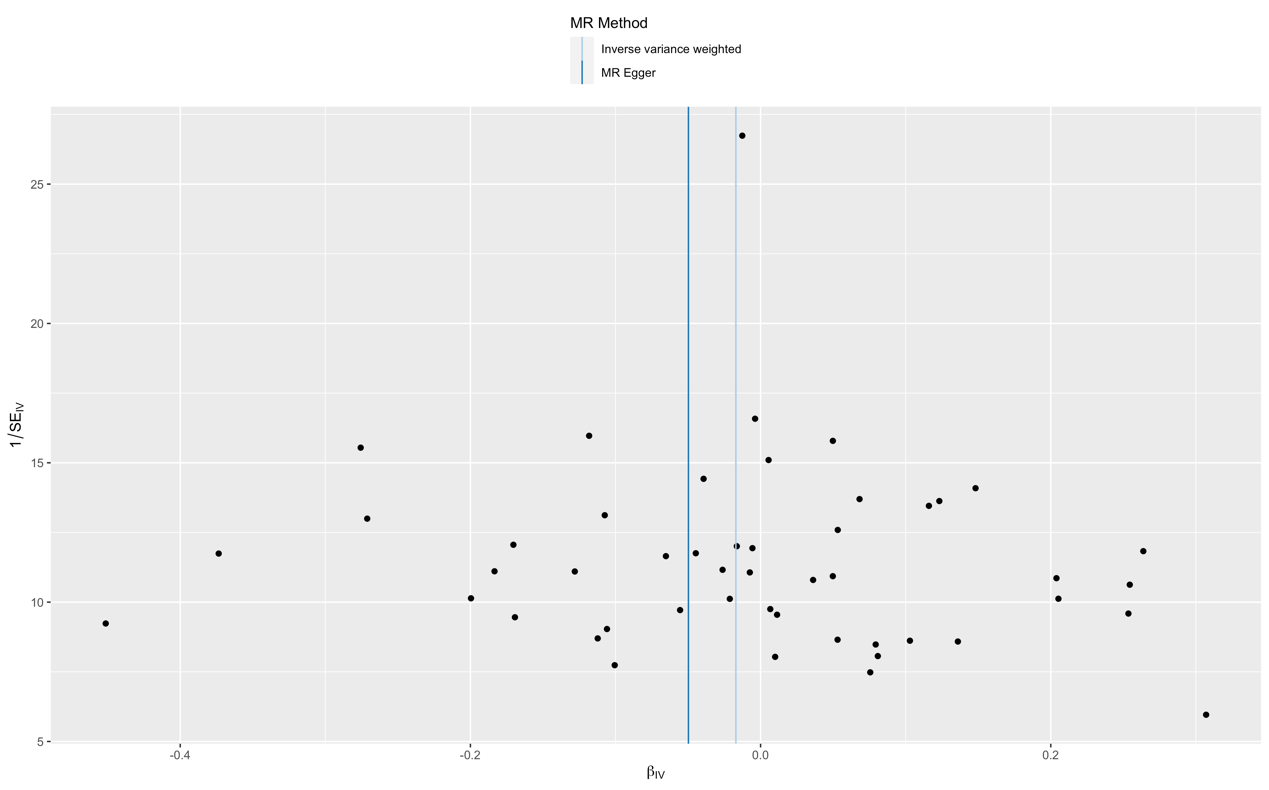


C. Funnel plot of causality between IBD and COPD.

**Osteoarthritis**


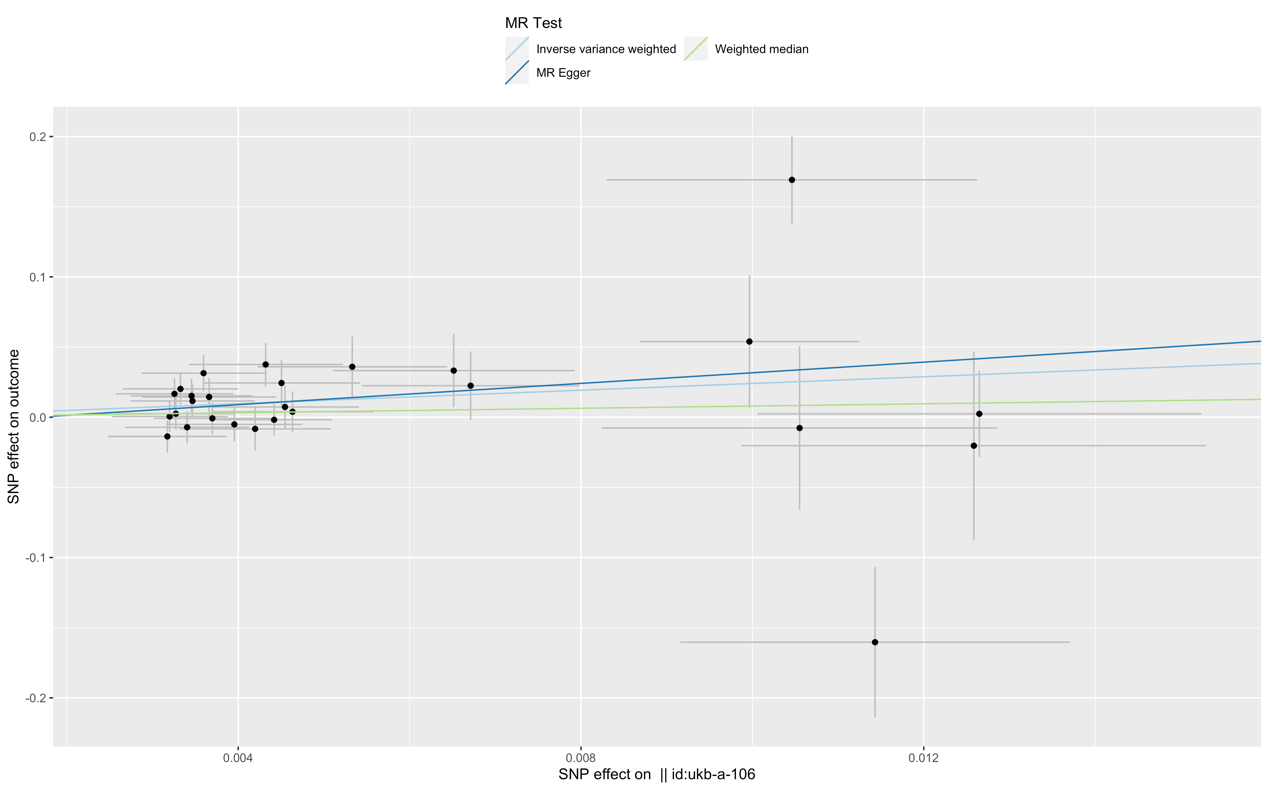


A. Scatter plot for casual effects of OA on COPD. SNP: single nucleotide polymorphism. The slope of each line represents an estimate of the effect of a different method using MR.


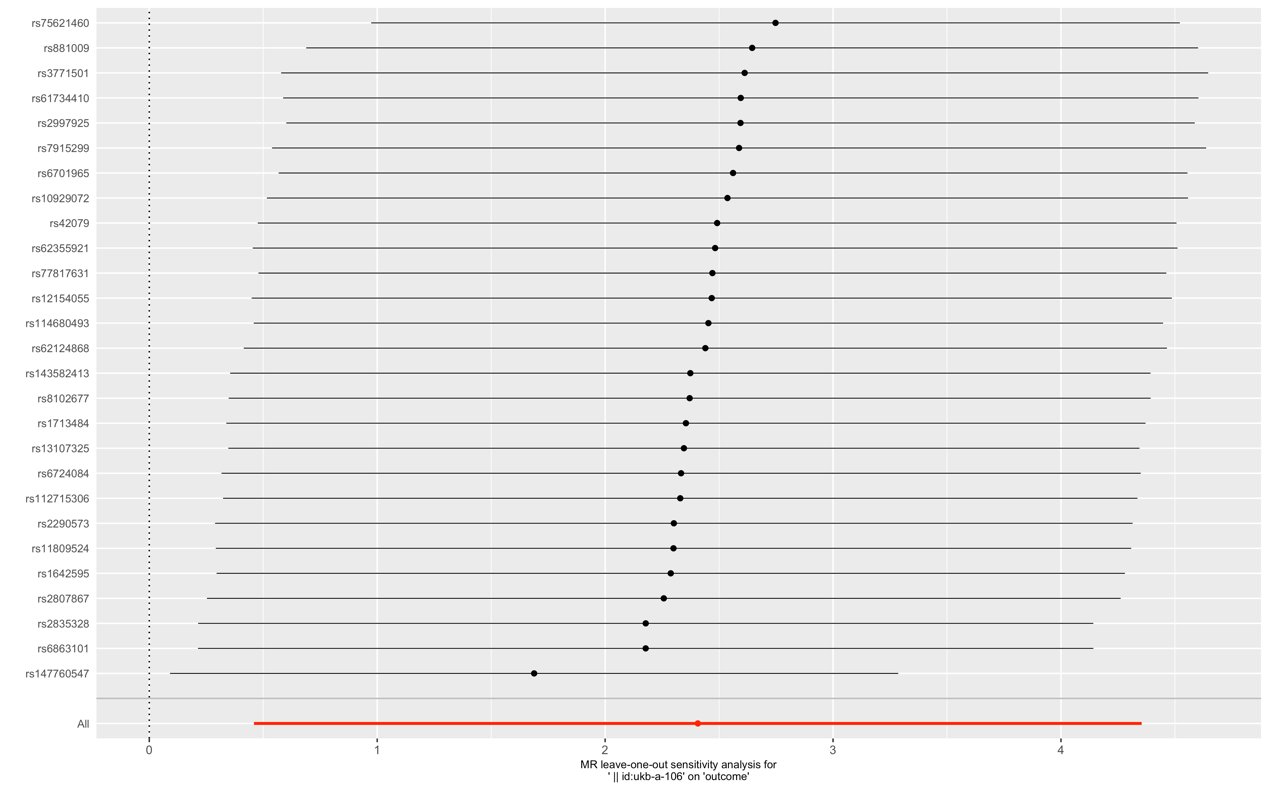


B. The leave-one-out sensitivity analysis assessed the causal association between OA and COPD.


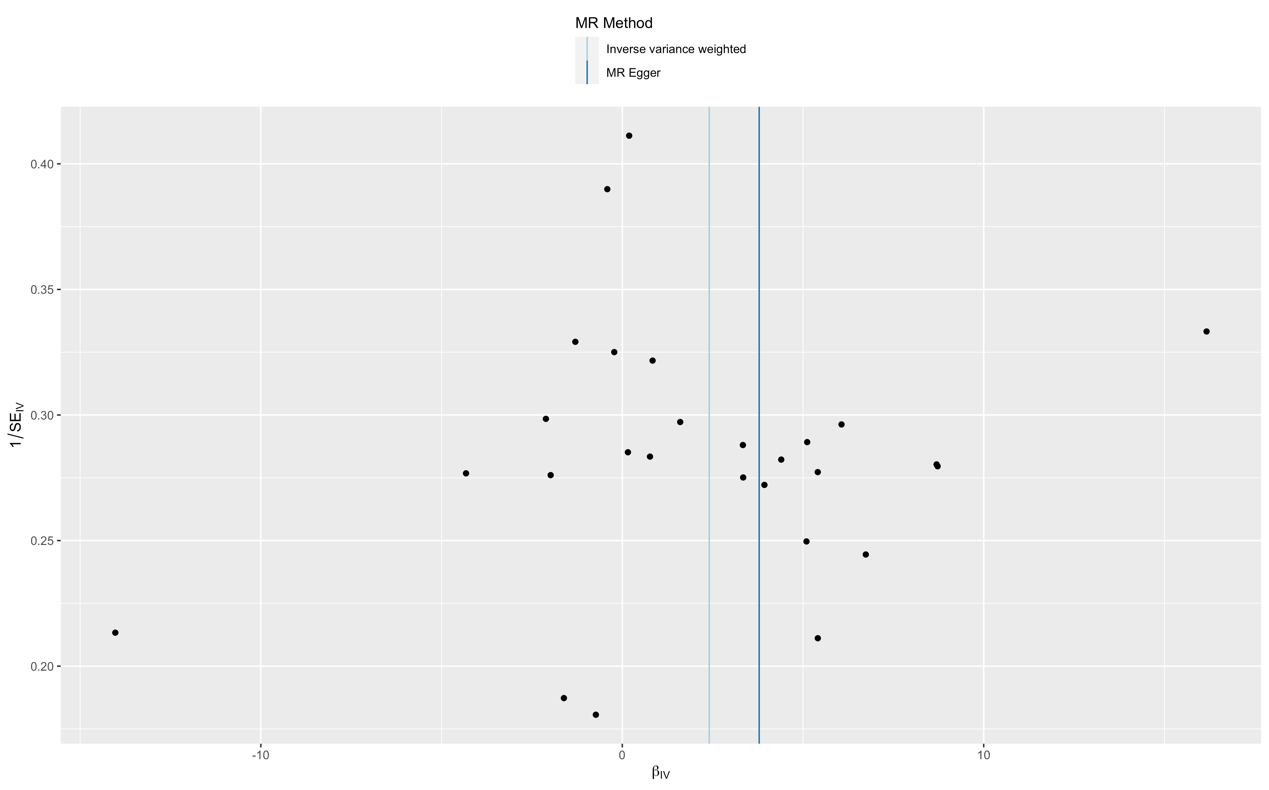


C. Funnel plot of causality between OA and COPD.
